# Metabolic characterization of human aqueous humor in the cataract progression after pars plana vitrectomy

**DOI:** 10.1186/s12886-018-0729-y

**Published:** 2018-02-27

**Authors:** Yinghong Ji, Xianfang Rong, Yi Lu

**Affiliations:** Department of Ophthalmology and Eye Institute, Eye and ENT Hospital of Fudan University, Key Laboratory of Myopia of State Health Ministry, and Key Laboratory of Visual Impairment and Restoration of Shanghai, No. 83 Fenyang Road, Shanghai, 200031 China

**Keywords:** Aqueous humor (AH), Metabolite-metabolite correlation, Metabolomics, Pars plana vitrectomy (PPV)

## Abstract

**Background:**

While pars plana vitrectomy (PPV) has become the third most commonly performed surgery in the world, it can also induce multiple post complications easily. Among them, cataract progression is the most frequent one that can lead to blindness eventually.

**Methods:**

To understand the underlying mechanisms of post PPV cataract progression, we performed comprehensive metabolic characterization of aqueous humor (AH) samples from 20 cataract patients (10 post PPV complication and 10 none PPV cataract) by a non-targeted metabolomic analysis using gas chromatography combined with time-of-flight mass spectrometer (GC/TOF MS).

**Results:**

A total of 263 metabolites were identified and eight of them are determined to be significantly different (VIP ≥ 1 and *p* ≤ 0.05) between post PPV group and none PPV control group. The significantly changed metabolites included glutaric acid and pelargonic acid that play key roles in the regulation of oxidative stress and inflammatory responses. Furthermore, we constructed a metabolic regulatory network in each group based on metabolite-metabolite correlations, which reveals key metabolic pathways and regulatory elements including amino acids and lipids metabolisms that are related to cataract progression.

**Conclusions:**

Altogether, this work discovered some potential metabolite biomarkers for post PPV cataract diagnostics, as well as casted some novel insights into the underlying mechanisms of cataract progression after PPV.

**Electronic supplementary material:**

The online version of this article (10.1186/s12886-018-0729-y) contains supplementary material, which is available to authorized users.

## Background

Pars plana vitrectomy (PPV) was first introduced about 35 years ago. The procedure is relatively safe, but unintended long-term complications may still occur that endangers complete visual rehabilitation of patients [1–3]. For example, cataract formation is known to be the most frequently happened complication of PPV, and more than 80% of patients develop cataract within 2 years after surgery [4]. Two types of cataract are most common after vitrectomy: posterior subcapsular cataract and nuclear sclerotic cataract. Nuclear sclerotic cataract progression after PPV has been known for many years, which may be caused by the deficiency of ascorbate, a powerful antioxidant [5].

Metabolomics analysis can provide comparative, semi-quantitative information of a large number of metabolites in biological samples from different groups at a specific time [6]. In recent years, this powerful –omics technology has been applied in ophthalmology researches such as diabetic retinopathy, glaucoma, and cataract progression, aiming to identify metabolic biomarkers and pathways that are important for understanding disease mechanisms and developing novel diagnosis and therapies [6–8]. Pushpot et al. performed metabolic profiling of serum and urine samples collected from patients with atrophic age-related macular degeneration (AMD) and neovascular AMD. They found that while arginine increases in neovascular AMD samples, glucose, lactate, glutamine and reduced glutathione decrease [9]. Mayordomo-Febrer et al. used high-resolution 1H NMR to analyze aqueous humor (AH) samples from control and glaucoma patients, and showed that levels of amino acids, carbohydrates, and lipids are all significantly altered after sodium hyaluronate injection series [7]. It was believed that these metabolic changes may play important roles in the pathogenesis of glaucoma.

AH is a very important intraocular fluid which is necessary for normal eye functions [10]. Knowledge gained from metabolic characterization of AH samples can be very helpful in advancing researches of many eye diseases. AH samples have been studied using nuclear magnetic resonance (NMR), liquid chromatography combined with mass spectrometry (LC-MS), and capillary electrophoresis combined with mass spectrometry (CE-MS), but to our knowledge, the metabolome for patients after PPV has never been determined using GC-MS technology [7, 10–12].

In this study, we analyzed AH samples from 20 patients using GC/TOF MS technology. Ten of the patients had received PPV and the other 10 did not. Significant metabolic variations were discovered between the two groups. Metabolite-metabolite correlation network in each group was also constructed, which reveals key regulatory pathways including amino acid and lipid metabolism pathways that are related to post PPV cataract progression. Altogether, the identified metabolites and regulatory metabolic pathways that changed significantly in the AH of patients after PPV may play important roles in the development of post PPV cataract.

## Methods

### Subjects

Totally 20 subjects were recruited for the present study: 10 post PPV complication and 10 none PPV cataract (Table 1). In the former group, a standard 23-gauge 3-port PPV was employed as the previous study, and the PPV surgery has been performed for more than half a year [13]. The intraoperative lens injuries during PPV were excluded. The other 10 controls had age-related cataract. Besides, all collected subjects met inclusion criteria, which had no history or slit-lamp evidence of ocular trauma, no use of systemic antimetabolites, corticosteroids, or immunosuppressants, and no unrelated ocular disease other than cataract. Furthermore, all patients signed the consent form, which was approved by the Ethics Committee of Eye & ENT Hospital of Fudan University, Shanghai, China.

**Table 1:** Summary of human aqueous humor samples

Group	Patient ID	Gender	Age range (Years old)	Axial length	LOCSIII
Controls	A36_1	Female	50–55	24.32	C3N3P3
	A37_1	Female	50–55	24.75	C2N2P5
	A38_1	Female	50–55	24.63	C3N4P2
	A39_1	Female	55–60	22.59	C3N3P2
	A40_1	Male	60–65	24.65	C2N3P2
	A41_1	Male	60–65	23.2	C3N2P4
	A42_1	Male	65–70	23.8	C3N3P3
	A43_1	Male	65–70	24.01	C4N5
	A44_1	Female	65–70	22.96	C4N5
	A45_1	Male	70–75	23.63	C5N4P2
Patients after pars plana vitrectomy	P1_1	Male	50–55	26.92	C2N5P3
	P2_1	Male	50–55	25.76	C2N4P2
	P3_1	Male	50–55	29.63	C3N5P4
	P4_1	Female	55–60	27.79	C2N3P3
	P5_1	Female	55–60	25.63	C3N3P2
	P6_1	Female	60–65	26.53	C3N4P3
	P7_1	Female	60–65	23.94	C3N4P3
	P8_1	Female	60–65	24.91	C2N3P2
	P9_1	Male	60–65	25.45	C2N3P2
	P10_1	Male	70–75	25.78	C3N4P3

### Sample extraction and preparation

Samples of AH were collected from Eye &ENT Hospital of Fudan University and extracted as the same with our previous study [14, 15]. After final centrifuging, all the samples were rapidly stored at − 80 °C until further analysis.

### GC/TOF MS analysis

The metabolic profiling for all 20 samples was performed as the same with our previous study [15]. After derivatization, the samples were analyzed by gas chromatograph (Agilent 7890A, Agilent, USA) combined with a Pegasus 4D time-of- flight mass spectrometer (LECO ChromaTOF PEGASUS 4D, LECO, USA). Meanwhile, mass spectrometry data were acquired with the m/z range of 20–600 and then mapped to spectra in the National Institute of Standards and Technology (NIST, http://www.nist.gov/index.html) and Fiehn databases as our previous report [15].

### Data analysis

Data normalization was firstly performed as previous studies [15–17]. Briefly, raw area counts of each compound were divided by its median value, while missing values (if any) were assumed to be below the limits of detection and were imputed with the observed minimum. The data after normalization step followed normal distribution, which was confirmed by SPSS 17.0 software. Mev (MultiExperiment Viewer) 4.8 was employed for K-Medians clustering analysis. Significant changed metabolites were determined in partial least squares discriminant analysis (PLS-DA) model, followed by independent t tests (SPSS 17.0 software) as other studies and our previous study [15–19]. Metabolites with both VIP (variable importance in the projection) values in PLS-DA model more than 1 and *p* values (in t tests) less than 0.05 were considered to be significant. The metabolite-metabolite correlation analysis was performed by using the R statistical software.

## Results

### AH samples collection

In the present study, these 20 samples were patients with moderate or even severe cataract, preparing for cataract surgery. They included 10 from controls of age-related cataract (ARC), and 10 from patients after PPV. The details for the 20 subjects including sex, age, and axial length were listed in Table 1. The average age of the patients after PPV was nearly 59, while the average age for the controls was about 60. Obviously, there was no statistical significance between those two groups for both sex and age. Furthermore, further binary logistic regression analysis based on the ratio C/(C + N + P) indicated the type between the two groups was significantly different (*p* ≤ 0.05). As a result, the present study emphasized on uncovering the underlying mechanism that patients after PPV easily develop nuclear and posterior capsular cataract instead of cortical cataract.

### Metabolic profiling of human AH

Taking advantage of a non-targeted metabolomic technology, we here employed GC/TOF MS to fully uncover AH metabolome. 263 metabolites in total were identified in those 20 AH samples including 10 patients after PPV and 10 patients for controls (Additional file 1: Table S1). The identified 263 metabolites contained 32 amino acids, 13 lipids, 44 carbohydrates, 6 nucleotides and other 168 biochemicals. Among those 168 metabolites, there were 39 named metabolites and 68 metabolites identified as analytes, and 61 metabolites determined to be unknown. Hence, the result here revealed 134 named biochemicals of the identified 263 metabolites, which involves in major metabolism pathways such as super pathway of amino acid, lipid, carbohydrate, nucleotide, etc. Importantly, as compared to previous studies, the result uncovered the broadest AH metabolome for human to date [7, 10–12, 15].

### Metabolomic study with patients after PPV and the controls

**Fig. 1:**
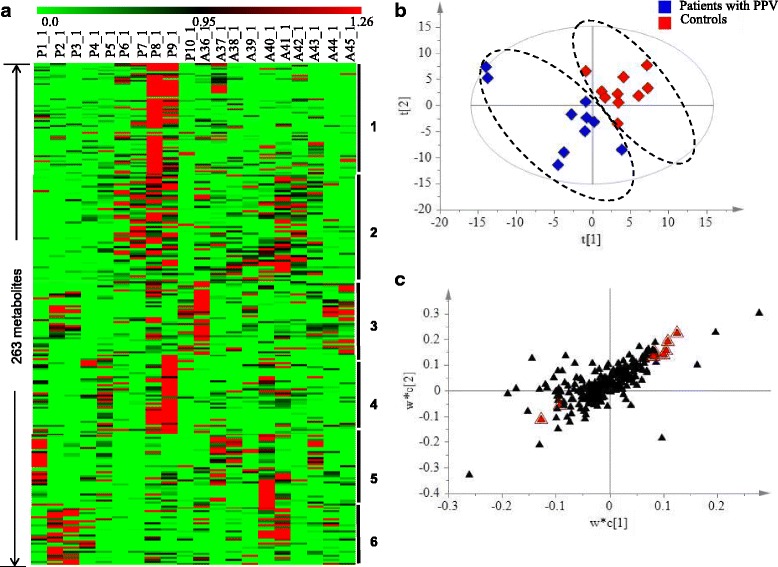
Metabolic variation between the controls and patients after PPV. **a** Heat map representation of 263 metabolites detected in 20 AH samples, showing 6 classes by clustering analysis. Each line represents one metabolite. The deeper the green color, the lowest its content in the AH sample; similarly, the deeper the red color, the highest its content in the AH sample. **b** PLS-DA score plot for the first two components (t[2] / t[1]) model for the controls and patients after PPV. **c** PLS-DA S loading plot for the two first components (w*c[2] / w*c[1]) for the controls and patients after PPV. Metabolites responsible for separation are labeled with red triangle

To review metabolic variation between the controls and patients after PPV, we performed clustering analysis by MEV 4.8, showing a plot of all the 263 biochemicals vs 20 samples, which were finally grouped into 6 classes (Fig. 1a). Obviously, the abundances of those 263 metabolites showed significant diversity across the 20 AH samples and more importantly, certain enriched metabolites seemed to be group-specific or individual-specific. For example in class 1, 60 of 67 metabolites appeared to be very high levels only in P8_1, while certain metabolites were enriched in patients after PPV including P1_1 (class 5), P9_1 (class 1), and P9_1 (class 4). Conversely, in class 3, 34 of 40 metabolites were abundant only in A36_1. Furthermore, several metabolites were specifically enriched in both two groups. For instance, certain metabolites in class 2 were enriched in P6_1, P7_1, P8_1, P9_1, A41_1, and A42_1.

The widely used supervised method, PLS-DA, was then employed for determining metabolic changes between the controls and patients after PPV. As shown in Fig. 1b, ten samples from patients after PPV were completely apart from those from the controls. Further statistical analysis including independent t tests showed that eight metabolites (Table 2 and Fig. 1c) were significantly different (*p* ≤ 0.05), responsible for the separation for those two group. These eight metabolites including 2 increased and 6 decreased biochemicals referred to 3 super pathways such as amino acid super pathway, lipid super pathway, and others. The only two increased metabolites were 3-(2-Hydroxyphenyl)propionic acid and unknown 029, whose ratio between patients after PPV and the controls were respectively 2.04 and 1.55. On the other hand, the left six decreased metabolites ranged from 0.34 to 0.70 folds including 4-acetamidobutyric acid 1, glutaric acid, and pelargonic acid.

**Table 2:** List of different metabolites between patients after PPV and the controls, responsible for the separation

Super Pathway	Biochemical Name	Retention time (minutes)	Ratio(PPV/ARC)^*^	*p* value
Amino acid	4-Acetamidobutyric acid 1	9.96	0.34	0.0347
Lipids	Glutaric Acid	9.31	0.43	0.0371
	Pelargonic acid	9.05	0.67	0.0173
Others	3-(2-Hydroxyphenyl)propionic acid	11.16	2.04	0.0019
	Analyte 389	14.16	0.59	0.0457
	unknown 029	9.64	1.55	0.0183
	unknown 037	10.39	0.70	0.0198
	unknown 056	13.85	0.60	0.0142

^*^ARC represented the controls

### Metabolic network variation based on metabolite-metabolite correlation

**Fig. 2:**
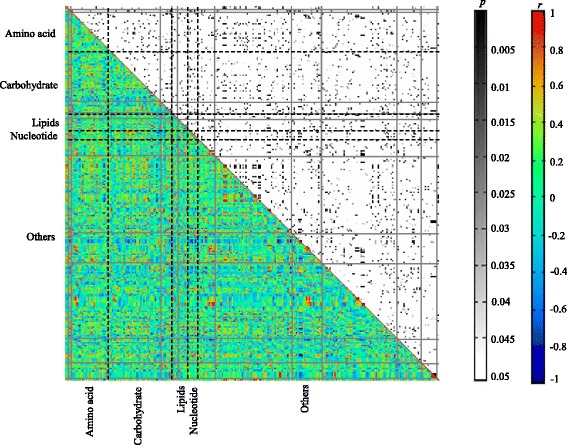
Metabolite-metabolite correlation analysis in the controls (The control group). Metabolites were shown in X and Y-axes, grouped by pathway information. Both *p* and *r* values of the correlations between every two metabolites were displayed in distinct colors

In order to uncover the regulatory of metabolic network in AH, we conducted metabolite-metabolite correlation analysis for both groups. As in the control group, there were a total of 31,626 associations, ranging from − 0.9769 for lysine and analyte 381 to 0.9988 for analyte 30 and analyte 32 (Fig. 2). Moreover, 1666 significant associations were then determined (r ≥ 0.7 & r ≤ − 0.7 and *p* ≤ 0.05), among which 1415 were positive associations while 251 were negative ones. Notably similar with our previous study in relation to high myopia, here amino acids also dominated the significant metabolite-metabolite associations (395 associations) including 54 negative ones [20]. All 20 standard amino acids except cysteine, arginine, glutamic acid, aspartic acid, histidine, threonine, and leucine had 150 significant associations, including 25 negative ones. Asparagine had 20 significant associations with only one negative correlation. Likewise, lysine had 20 significant associations but including 18 negative ones. Moreover, 43 carbohydrates had 402 significant associations including 69 negative ones. Among them, fructose had 8 positive associations and 3 negative ones. Similarly, 12 of 13 lipids had 103 significant associations with six negative ones. Especially, 19 associations were connected with linoleic acid methyl ester, which were all positive.

**Fig. 3:**
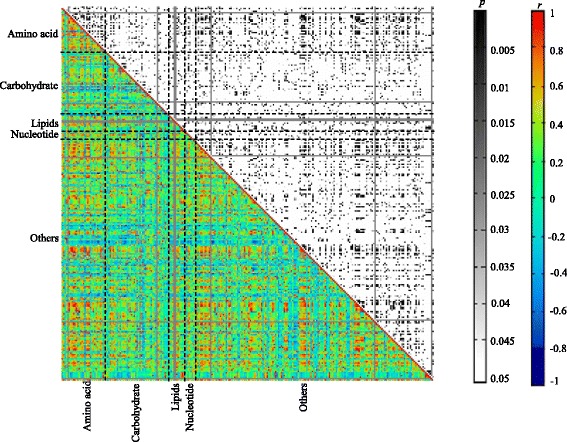
Metabolite-metabolite correlation analysis in patients after PPV (The experiment group). Metabolites were shown in X and Y-axes, grouped by pathway information. Both *p* and *r* values of the correlations between every two metabolites were displayed in distinct colors

Likewise based on the identified 263 metabolites in patients after PPV, metabolic network was also constructed to analyze the regulatory. As shown in Fig. 3, a total of 32,640 associations were identified, ranging from − 0.92452 for purine riboside and analyte 48 to 0.99997 for analyte 363 and analyte 402.Moreover, there were 3575 significant associations (r ≥ 0.7 & r ≤ − 0.7 and *p* ≤ 0.05): 3482 positive associations and 93 negative ones. Likewise in patients after PPV, amino acids still dominated the significant associations as the same with control group. A total of 1044 significant associations (including 22 negative associations) were determined for amino acids. Among them, all 20 standard amino acids except glutamic acid, cysteine, arginine, leucine, aspartic acid, histidine, and threonine had 493 significant associations including three negative associations. All the negative associations were associated with proline. Notably, 71, 67, and 64 associations were respectively associated with serine 1, asparagine 4, and lysine. Moreover, 42 carbohydrates had 634 significant associations including 40 negative associations. Among them, there were 23 positive associations for fructose. 11 of 13 lipids had 208 significant associations including four negative ones, and 17 associations were positively associated with linoleic acid methyl ester.

## Discussion

Cataract is one of the highest frequently eye diseases, which can eventually lead to blindness. It’s possible now to cure cataract through surgery for removing the diseased lens and replacing by a clear one, but it is difficult to efficiently control cataract in the nearby future. Hence, it is very important for us to reveal the underlying mechanism of the formation of cataract. We previously reported the metabolic characterization of human aqueous humor associated with high myopia [15]. The results showed considerable metabolic variations including both metabolite abundances and the metabolite-metabolite associations in the development of nuclear and posterior capsular cataract due to high myopia. Similarly, PPV have long been reported to be associated with the development of nuclear and posterior capsular cataract. However, we believed patients after PPV may easier develop cataract than those with high myopia, since full of the vitreous cavity in patients after PPV is aqueous humor without normal function acting as a gel and interconnected meshwork. In order to reveal the underlying mechanism in the cataract progression after PPV, in the present study by using the same technology, we collected 20 aqueous humor samples from 20 cataract patients (10 post PPV complication and 10 none PPV cataract for metabolomics analysis. It should be pointed that both two groups of patients were with cataract, while patients in the control group had age-related cataract. As a result, the study here might emphasis on revealing metabolic changes at progressive stage of cataract development for patients after PPV other than pathogenesis of cataract at early time.

Obviously, significant different metabolic characterization could be observed in patients after PPV when compared with those in patients with high myopia, which demonstrated that there is a different underlying mechanism in the cataract progression after PPV. Firstly based on the same metabolomics technology and data analysis methods, totally 263 metabolites were identified in this study, which was larger than that in our previous study on high myopia. Secondly, very few significantly changed metabolites were determined between patients after PPV and the controls. Here eight metabolites were significantly different (*p* ≤ 0.05), responsible for the separation for these two groups, while 29 metabolites were determined to be significantly different in patients with high myopia. Finally, more (significant) correlation analyses were identified in the present study. Especially, there were a total of 1044 significant associations (including 22 negative associations) between amino acids and metabolites in other super pathways or among amino acids in patients after PPV, while only 703 significant associations for amino acids in patients with high myopia.

As a matter of fact, there has been already a nutrient theory of cataractogenesis as a function of altered aqueous fluid dynamics [5]. For example, Chung et al. (2001) observed that after removal of part of the vitreous, nuclear cataract may be resulted from altered lens metabolism [20]. It is believed that powerful antioxidant ascorbate is deficient in the vitreous cavity after vitrectomy, which may be one of the factors to trigger the development of cataract. Moreover, another a major mechanism resulting in senile nuclear cataract is posttranslational modification of lens crystallins through glycation, which includes glucose and the oxidative products of ascorbic acid: dehydroascorbate, 2,3 diketogulonic acid, xylosone, and threose [5]. In the present study, eight metabolites of 263 detected metabolites were found to be significantly changed in patients after PPV, including one amino acid and two lipids. These significantly changed metabolites may play very important roles in cataract development, which are likely to be potential biomarkers for cataract diagnostics. For example, glutaric acid was reported to be involved in the regulation of oxidative stress, which is an initiating factor for the progression of maturity onset cataract [21]. So the change of the level of glutaric acid in patients after PPV may help explain the progression of cataract after PPV [22]. Meanwhile, inflammation has also been reported to induce cataract (especially for PSC) after vitrectomy [5]. Here we found a lower level of pelargonic acid in patients after PPV, which was reported to have antifungal property [23]. Thus the change of this metabolite may involve in inflammatory response that contributes to cataract progression.

The metabolite-metabolite correlation analysis is supposed to dissect putative key regulatory elements or pathways for metabolism regulation, and proved to be helpful for discovering novel pathways [15, 17]. Correlation analysis of metabolomics data in our study indicated much more (significant) correlation analyses for amino acids and lipids in patients after PPV than those in the control group. The results here suggested both the metabolism of amino acids and lipids may play significant roles in the progression of cataract after PPV [5]. Usually, more active of amino acids or proteins could be observed in the development of cataract, which has been reported by other studies and our previous studies [14, 15]. And especially in the present study among the standard amino acids, serine 1 accounted for the most associations in patient after PPV, which was reported to be associated with the formation of human age-related conditions such as cataract [24].

## Conclusions

In conclusion, taking advantage of an unbiased technology GC-TOF-MS, we fully showed the metabolic characterization of AH in patients after PPV. More importantly, the significant metabolic variation including metabolite abundances and metabolic networks in PPV revealed key regulatory elements or pathways especially referred to amino acids and lipids metabolism in relation to cataract progression. The results here would extend our understanding on the underlying mechanism and may provide potential biomarkers for cataract diagnostics. However in the present study, there were still some deficiencies including the small number of samples and proper samples for control group due to ethical issues. At the same time, further studies with a great number of samples and much more effort are supposed to be done for validating the potential biomarkers for cataract diagnostics. Additionally in combination with other data/technologies including genomics and proteomics, these findings on metabolic changes need further explore to fully reveal the underlying mechanism in the cataract progression after PPV.

## Additional file

Additional file 1: **Table S1.** List of 263 detected metabolites with some important properties including CAS, KEGG and PubChem entry numbers. (XLS 58 kb)

## Abbreviations

AH: Aqueous humor
AMD: Age-related macular degeneration
ARC: Age-related cataract
BSS: Balanced salt solution
CE-MS: Capillary electrophoresis–mass spectrometry
CME: Cystoid macular edema
ERM: Epiretinal membrane
GC/TOF MS: Gas chromatography coupled to time-of-flight mass spectrometer
GC-MS: Gas chromatography-mass spectrometry
LC-MS: Liquid chromatography-mass spectrometry
LOCSIII: Lens opacities classification system III
NMR: Nuclear magnetic resonance
PCED: Persistent corneal epithelial defects
PLS-DA: Partial least squares discriminant analysis
PPV: Pars plana vitrectomy
RRD: Rhegmatogenous retinal detachment
VIP: Variable importance in the projection
